# Culex genome is not just another genome for comparative genomics

**DOI:** 10.1186/1756-3305-5-63

**Published:** 2012-03-30

**Authors:** BP Niranjan Reddy, Pierrick Labbé, Vincent Corbel

**Affiliations:** 1 School of Studies in Biotechnology, Jiwaji University, Gwalior, MP 474 002, India; 2 Laboratoire Génétique et Environnement, CNRS UMR 5554, Institut des Sciences de l'Evolution, Université Montpellier 2, Place E. Bataillon, 34095 Montpellier cedex 05, France; 3 Institut de Recherche pour le Développement (IRD), Maladies Infectieuses et Vecteurs, Ecologie, Génétique, Evolution et Contrôle (MIVEGEC, UM1-CNRS 5290-IRD 224), Centre de Recherches Entomologiques de Cotonou (CREC), 06 BP 2604 Cotonou, Bénin

## Abstract

Formal publication of the Culex genome sequence has closed the human disease vector triangle by meeting the Anopheles gambiae and Aedes aegypti genome sequences. Compared to these other mosquitoes, Culex quinquefasciatus possesses many specific hallmark characteristics, and may thus provide different angles for research which ultimately leads to a practical solution for controlling the ever increasing burden of insect-vector-borne diseases around the globe. We argue the special importance of the cosmopolitan species- Culex genome sequence by invoking many interesting questions and the possible of potential of the Culex genome to answer those.

## *Culex quinquefasciatus*: a disease vector for multiple mosquito-borne pathogens

*Drosophila melanogaster *genome sequence was the first insect genome published in 2000 with an aim to understand various aspects of the insect biology [1] including the biological aspects that are important for disease vectors. Progressing through the post-genomic times has made us realize that many of the biological processes are species-specific and thus *Drosophila*, a species that could not host parasites and is not a targeted human disease vector for control, is probably not the most suitable model to study the vector borne diseases [2,3]. Hence, in 2002, the first haematophagous insect and a principal malaria vector, *Anopheles gambiae *genome sequence was unveiled to get closer to a real-time 'disease-vector' scenario [2]. A paradigm shift in vector control research methodology was evident after the gambiae genome sequence, and this feature has further encouraged the scientific community to pursue the genome sequence of another important mosquito vector - *Aedes aegypti*, which hosts arboviral diseases like dengue and Chikungunya. The primary objective of the *Aedes *genome sequencing was to understand the host-pathogen (virus) interactions in general, and the dengue pathogenesis and prognosis in particular [4]. Due to availability of mosquito(s) genome sequence information coupled with a wealth of specialized comparative genomics - the bioinformatic tool box has paved the way of vector control research towards finding a practical solution for vector control through deciphering various elusive processes that are specific to diseases vectors. Albeit the exceptional amount of molecular biological research on vectors and pathogens produced after the publication of genome sequences, which is evident from the amount and nature of published research, indeed there is still a great amount of work that needs to be done to understand the vector biology at a molecular level, such that the information could be employed for a practical application of the knowledge for disease management. Under these circumstances, in addition to the existing *An. gambiae *and *Ae. aegypti *genome sequences, how will another disease vector genome sequence from *Culex quinquefasciatus *[5] help to fill the knowledge gap in vector biology? Here, we emphasize the importance of *Cx. quinquefasciatus *genome sequence in different facets, and defend that the "*Culex *genome sequence is more than just another genome for comparative genomics".

*Cx. quinquefasciatus *is an important member of the *Cx. pipiens *complex that can transmit a wide variety of pathogens of medical and veterinary importance [5,6]. It can transmit viral diseases such as West Nile encephalitis, Eastern equine encephalitis, Venezuelan equine encephalitis, Japanese encephalitis, St. Louis encephalitis, Ross River encephalitis, Murray Valley encephalitis, Rift valley fever, and the nematode disease, lymphatic filariasis. It can also transmit the avian malaria parasite, *Plasmodium relictum *to birds. The *Culex *species have a cosmopolitan distribution [7-9] and are primarily responsible for mosquito nuisance in tropical and sub-tropical urban/peri-urban settings around the globe.

## Species-specific hallmark characteristics of *Culex *species

Resurgence and spread of vector borne diseases (VBDs) is becoming a serious public health problem throughout the world which is obvious from a burgeoning shift in the number of cases and the ever changing dynamics of epidemiology of the disease(s) incidence. The appearance, establishment and spread of VBDs are dependent on environmental, social-demographic and climatic factors. The availability of the *Culex *genome sequence has opened up new opportunities to look into various disease and vector control aspects [5], for example, multiple parasites-vector interactions, the role of environmental pollutants in strengthening the detoxification system, and diagnosis, tracking and modeling of the development of insecticide resistance (IR), etc. In comparison to *An. gambiae *and *Ae. aegypti *genetic make-up, this species has indeed special characteristics; (a) it is widely distributed all over the world, both in temperate and tropical regions [10], (b) it can harbor a large variety of viral, nematode, and protozoan human disease parasites [5,7], (c) it can oviposit and develop in a wide range of polluted and non-polluted larval habitats, (d) it can target diverse species (mammals including humans or birds) for their blood-meal, based on opportunity, (e) in a sharp contrast and as an exception to the thumb-rule of haematophagous insects that a blood-meal is indispensable for egg laying, some *Culex *can lay the first round of eggs without it [11], and (f) *Culex *resistance to various insecticides used in the vector control has been deeply investigated throughout half of a century and thus it represents one of the iconic models of adaptation by means of natural selection [12-14]. Interestingly, the preliminary analysis of *Cx. quinquefasciatus *genome revealed a repertoire of 18,883 protein-coding genes and this number is 22% and 52% larger than that of the *Ae. aegypti *and *An. gambiae *gene repertoires, respectively. Bartholomay *et al. *[7] show that these expansions are primarily due to multiple gene-family expansions that include olfactory and gustatory receptors, salivary gland genes, and genes associated with xenobiotic detoxification. It is thus important to understand how these relatively massive increases in specific gene copy number interfere with the blood feeding preferences, transmission of pathogens, and vector population adaptation to various polluted environments.

## A model for studying the detoxification system in mosquitoes

Despite the availability of a wealth of information on insecticide resistance (IR) genomics in public domains, there is still a gap in understanding inter-connections between the various molecular networks that are involved in the development of IR; in this respect having the full *Culex *genome sequence can be a decisive factor for future progress in this area. One of the interesting questions that can be answered by using the *Culex *genome is the impact of environmental pollution on the selection of IR: this species' habitat preference for polluted water will deeply impact; (a) its survival and longevity, and thus its vectorial capacity to transmit disease causing pathogens, (b) its immune system, and thus its ability to harbor different varieties of pathogens, and (c) the origin and spread of new IR mutations, and occurrence of multiple resistance mechanisms in a single species. IR can be caused due to the biology of the mosquito which makes them avoid the insecticide treated surfaces, involvement of detoxification enzymes (cytochrome P450s, glutathione-S-transferases, and choline/carboxylesterases), target site insensitivity causes due to single or multiple point mutations, and a change in the physiology of the mosquito. As a matter of fact all of these mechanisms are associated with a cost to the organism, and hence the occurrence of multiple insecticide resistance mechanisms conjointly in a single mosquito population is a matter of thorough investigation; keeping in view of the vector control field operations for disease containment without prompting the development of mosquito species' resistance to old or newly introduced insecticides.

As a recent adaptation, IR is often associated with a fitness cost in mosquitoes. Due to this, absence of insecticide selection pressure will reduce the frequency of IR alleles pertaining to this insecticide. In contrary to this natural phenomenon, several examples show that the *Culex *species have maintained multiple insecticide resistance mechanisms despite the absence of insecticides that are specifically used to target these species. For this, the species needs a constant maintenance of IR cellular factors. This is where *Culex *can act as a model to solve questions pertaining to the multiple IR mechanisms and cross-talk between them to give an optimized cost-effective response to xenobiotics/insecticides. In this context, constant up-regulation of detoxification enzymes or other IR causing factors in *Culex *due to their larval habitat choices is an important subject that could be investigated by genomic and transcriptomic studies. A comprehensive study performed by Poupardin *et al. *[15] show that in *Ae. aegypti *cross induction of resistance to unchallenged insecticides is possible given a prior exposure to a mixture of classes of xenobiotics through the up-regulation detoxification enzymes.

A prior and thorough understanding of the impact of co-existence of multiple resistance mechanisms is necessary before any introduction of new insecticide combinations or molecules [16]. For example, the pleiotropy and/or epistasis interactions of different resistance mechanisms can modify their respective dynamics: it has been shown that the presence of knock-down resistance (*kdr*) allele in *Culex *can reduce the cost of the acetylcholinesterase-1 (*ace-1*) resistance allele, thus facilitating its spread in pyrethroid-resistant populations [17]. It is frequently observed in field populations of *Culex *spp. that they exhibit resistance to multiple insecticides [18], and many cross-resistance mechanisms are known to be conferred by detoxification enzyme alleles and target gene mutations like *kdr *and *ace1 *(see for a review [14]). The present genome sequence may offer a chance to understand these patterns of cross-resistance and various pathways associated. With respect to the IR, breeding preferences of *Culex *species could strengthen the detoxification system against new insecticides: for instance, the presence of chemical analogs in the larval breeding habitat of a mosquito could prepare their detoxification system against insecticides to which the species might never have experienced before. An example for this proposition can be drawn out from our recent observations. In India, *Cx. quinquefasciatus *mosquitoes revealed multiple IR in two urban localities, namely Raipur District, Chhattisgarh State, and Nadiad District, Gujarat State (for susceptibility data table see [19]). Interestingly, the species has shown high levels of resistance to DDT, malathion, and bendiocarb, and to a lesser extent to deltamethrin. These insecticides have never been used against *Culex *before, which could suggest a role of larval habitats and local environmental conditions (e.g. agriculture) on the insect resistance to various public health pesticides.

To date, through molecular and functional genomics studies on *An. gambiae *and *Ae. aegypti *it is known that a small portion of the detoxification enzymes' repertoire is responsible for the insecticide resistance phenotype [20]. A preliminary analysis on *Culex *detoxification systems revealed the presence of a total of 268 detoxification genes [21](Reddy and Raghavendra, unpublished results). However, given the fact that mosquitoes might get exposed to numerous varieties of xenobiotics during their complex life cycle, how these detoxifying enzymes can help to protect the mosquito against insecticides needs to be addressed. Finally, extensive studies using classical genetics and insecticide monitoring tools on *Culex *species have shown that passive migration plays an important role in spreading the IR alleles into susceptible areas. *Culex *is one of the three species that laid foundations to the present day concepts in IR, along with *Lucilia cuprina *and *Myzus persicae *[10]. The present availability of *Culex *genome sequence may assist in fine-tuning and fostering the understanding of the role of geographical barriers and of the different regimes of pest management strategies currently in application in different countries on the spread of IR alleles within and across different continents.

## A model for vector-pathogen interactions and understanding incipient speciation

The *Culex *genome annotation revealed 20-30% expansion in the immune responsive genes as compared with the *An. gambiae *(380) and *Ae. aegypti *(417) [7], suggesting a direct relationship between the number of immune genes and the capacity of the species to host multiple pathogens. In support of this hypothesis, the pathogenomic analysis of *Culex *genome by Bartholomay *et. al. *[7] using microarray has revealed that the immune gene repertoire in *Culex *is expanded in response to its plasticity to adapt to diverse habitats. This implies that breeding preference habitat of *Culex *species could be exploited to study the effect of a prior exposure of a species to a myriad of classes of microbes that exist in their larval growing habitat to understand the possible influences, if any, on the capability of a species to host diverse pathogens. Moreover, resistance genes can in turn impact on the spread of various parasites vectored by *Culex *[22]. Nonetheless, *Culex *mosquitoes are natural hosts to *Wolbachia *bacteria, which are found to effectively contain the viral pathogen multiplication (dengue, Chikungunya, West Nile virus, etc.) in mosquitoes [23,24]. *Wolbachia *acts as a reproductive parasite in mosquitoes and as an obligate symbiont in filarial nematodes [25]. Interestingly, *Culex *can host both *Wolbachia *and filarial nematodes, this unique feature of the species may provide an opportunity to understand vector-pathogen interactions. These aspects could be investigated more deeply thanks to the *Culex *genome availability, which may ultimately lead to elucidating the candidate elements in the *Culex*-*Wolbachia *relationship to improve laboratory manipulation of *Wolbachia *in the mosquitoes for their cost-effectiveness for easy propagation and spread in the wild for population replacement strategy of vector control. Last but not the least, the *Cx. pipiens *complex itself is the most controversial phylogenetic group in the *Culex *genus. Using comparative genomic studies, it may be now possible to improve our present understanding about incipient speciation in this species complex.

## Conclusion

Publication of the *Culex *genome sequence is a boon to study vector biology, host-parasite interactions, insecticide resistance, and evolutionary biology that are all merging at some point to answer a common question about "what is to be done to 'control' vector-borne diseases?" Through its unique features, the *Culex *genome could help in answering many questions, and thus may help in designing efficient vector control strategies.

## Competing interests

The authors declare that they have no competing interests.

## Authors' contributions

NR, VC, PL conceptualized the idea and NR has prepared the initial draft of the manuscript. VC and PL were involved in subsequent correction of the MS and all authors approved the final version of manuscript.
